# Examining Negative Emotional Symptoms and Psychological Wellbeing of Australian Sport Officials

**DOI:** 10.3390/ijerph17218265

**Published:** 2020-11-09

**Authors:** Fraser Carson, Natalie Dynon, Joe Santoro, Peter Kremer

**Affiliations:** 1 Department of Sport and Exercise Science, LUNEX International University of Health, Exercise and Sports, Differdange, 4671 Luxembourg, Luxembourg; 2 Centre for Sport Research, Deakin University, Geelong 3220, Australia; ndynon@deakin.edu.au (N.D.); j.santoro@deakin.edu.au (J.S.); peter.kremer@deakin.edu.au (P.K.)

**Keywords:** mental health, depression, anxiety, stress, workload, control

## Abstract

Sports officials are exposed to numerous performance and personal stressors, however little is known about their mental health and psychological wellbeing. This study investigated levels of mental health and psychological wellbeing of sports officials in Australia, and the demographic, officiating, and workplace factors associated with these outcomes. An online survey consisting of demographic and officiating questions, and measures of work engagement, mental health and psychological wellbeing was completed by 317 officials. A negative emotional symptoms score was computed. Associations between key demographic, officiating, and workplace factors with negative emotional symptoms and psychological wellbeing were assessed using univariate and multivariate analyses. Officials who were younger, not in a committed relationship, having lower levels of education, and less officiating experience reported higher levels of negative emotional symptoms, while males, older than 50 years, in a committed relationship and more officiating experience had higher levels of psychological wellbeing. The ability to self-manage workload and demonstrate professional autonomy were strongly associated with negative emotional symptoms and psychological wellbeing. Officials reported high negative emotional symptoms, but also high levels of psychological wellbeing. The ability to manage workload and to express professional autonomy are important determinants of mental health and wellbeing levels of sports officials.

## 1. Introduction

Sport officials face numerous challenges associated with their role. They have unique responsibilities and perform a multitude of activities within the uncontrollable, unpredictable, complex and nonlinear nature of sport. Officials often receive minimal instrumental support for their role and face public scrutiny, hostility, physical and verbal abuse, and even violence. These conditions may impact their mental health (MH) and psychological wellbeing (PWB) which may, in part, contribute to the attrition of sports officials [1]. MH and PWB are acknowledged to be separate, albeit related, constructs [2]. Previous research has reported on levels of MH and PWB for athletes but there is limited research for other sectors of the sport workforce. Recognising the lack of research on MH and PWB in sports officials, Gorczynski and Webb [3] called for a greater research agenda in order to improve the dissemination of evidence-based MH interventions for this sector.

The role of the sport official is primarily to uphold the integrity of the sport [4]. It involves both on-field and off-field responsibilities which include enforcing rules, standardizing competitions and keeping sporting contests safe for all participants involved [5]. The responsibilities range from stationary sole arbitrator (e.g., tennis umpire) to more actively involved team of officials (e.g., basketball referees) [6]. The role requires an ability to perform under pressure, focus their attention on specific components, and cope with adverse situations that can occur [7]. At all levels officials may experience abuse and threats [8] and this physical and psychological intimidation is a major cause of stress for officials [9]. Currently the direct impact of these on officials’ MH and PWB is unknown, but these are a major concern that require further research [3].

MH is defined as ‘a state of well-being in which every individual realizes his or her own potential, can cope with the normal stresses of life, can work productively and fruitfully, and is able to make a contribution to her or his community’ [10], while PWB refers to the balance between an individual’s positive and negative emotions and their overall life satisfaction [11]. Within this study the two continua model of mental illness and health [12] was applied, allowing MH and PWB to be examined as related but distinct dimensions. One continuum of the model relates to the presence or absence of mental health and the second continuum related to the presence or absence of mental illness. Bos et al. [13] identified that while higher levels of negative emotional symptoms may be present, an individual may still rate high in PWB. Reardon et al. [14] recommended assessing PWB alongside MH, as PWB is associated with more adaptive coping and more effective management of stress [15]. Applying the socio-ecological model [16], multiple factors may influence the MH and PWB of sports officials, including individual (e.g., age, experience) and workplace factors (e.g., workload, control). Studies have identified that as individuals age they are more likely to prioritise PWB than strive for career advancement [17], and older individuals are more autonomous in behaviour [18]. Experience in a role has the ability to enhance resilience and encourage better MH and PWB [19] and has been noted to decrease emotional exhaustion [20]. Self-efficacy increases with experience, which can act as a moderator against stress [21]. Higher levels of anxiety have also been reported for less experienced officials [22]. Higher levels of education are also associated with better MH and PWB [23]. Similarly, the benefits of being in a committed relationship is associated with less depressive symptoms and higher PWB compared to those not in such relationship [24], with social support important for promoting PWB [25].

Workplace factors that can influence MH and PWB are workload and control [26]. An inability to effectively manage workload increases the potential for MH issues [26], while a lack of professional autonomy is associated with poorer PWB [27]. This is increased when individuals work under sustained demands [28], which is the case for sports officials working across a season [29]. Gouttebarge et al. [30] reported a 4% increase in anxiety and depression levels for officials across a season. The balance between officiating and personal commitments is a known challenge for officials [31]. A lack of organisational support can also contribute to these negative factors and officials have requested more specific support measures [30]. Kilic et al. [21] posit that limited satisfaction with the support received was associated with lower PWB for officials. Occupational psychology literature in frontline workers supports the inclusion of conflict management training to assist with the management of verbally aggressive customers [32]. A potentially unique impact on their MH and PWB reported by officials is specific performance assessment (i.e., making an incorrect call) [33], particularly when under constant scrutiny from athletes, coaches and spectators. High demand for performance can negatively affect PWB [34], with Johansen and Haugen [22] reporting those officiating at higher levels of competition exposed to greater MH risks.

There is limited research currently related to MH and PWB in sport. This research has focused on specific sporting groups, primarily athletes [35] and coaches [36], with minimal attention to others from the wider sport workforce and in particular sport officials. Therefore, the aim of this study was to examine the MH and PWB in Australian sport officials participating at all performance levels, and the individual and workplace factors associated with these states. Specifically, the following hypotheses were assessed:

**Hypotheses** **1.** *Older officials will experience lower levels of negative emotional symptoms and higher levels of PWB.*

**Hypotheses** **2.** *Officials in a committed relationship will experience lower levels of negative emotional symptoms and higher levels of PWB.*

**Hypotheses** **3.** *A higher ability to manage workload will be associated with lower levels of negative emotional symptoms and higher levels of PWB.*

**Hypotheses** **4.** *Greater officiating autonomy (control) will be associated with lower negative psychological symptoms and higher levels of PWB.*

## 2. Materials and Methods

### 2.1. Sample and Recruitment

Managers of 33 local, state and national sport organisations and official associations within Australia were contacted via email with details about background to the study and a link to the online survey. These organisations and associations provided a plain language statement and the survey link to their registered officiating members. In order to be eligible to complete the survey, participants had to be 18 years of age or older and actively involved as a sports official. A specific population size is difficult to establish due to a lack of availability of registered and active sports official numbers. However, collectively it was estimated ~3500 officials were associated with the 33 organisations.

### 2.2. Instruments and Measures

An online survey was used to obtain demographic and officiating information as well as measures of work engagement, MH and PWB. Demographic questions included gender, age (years; 18–20, 21–25; 26–30; 31–35; 36–40; 41–45; 45–50; ≥51) highest level of education (high school; degree; post-graduate; other), and marital status (married/couple; single; divorced/widowed/separated; committed relationship). Officiating questions included: ‘what sport/s do you officiate in?’; ‘what level of competition do you usually officiate in?’ (local/community; state; national; international); ‘what age group/s do you usually officiate?’ (children/youth; adult/senior; both); ‘what gender group/s do you usually officiate?’ (male; female; both); ‘what is the nature of your (officiating) employment’ (volunteer; paid); ‘what is your (officiating) paid employment status?’ (part-time; full-time; casual); and ‘how many years have you been an official for?’ (years; 0–1, 2–3, 4–5, 6–7, 8–9, ≥10). See Table S1 (Supplementary Materials).

#### 2.2.1. Work Engagement

The Areas of Worklife Scale (AWS) [26] was used to measure the positive and negative work and homelife areas. It consists of 28 items that are reflective of six dimensions: (1) workload—the ability of manage job demands (5 items; i.e., ‘I have so much work to do on the job that it takes me away from my personal interests’); (2) control—the capacity perceived by individuals to impact the choices involved in their work, to exercise personal autonomy and access to resources with the end goal of finishing their work in mind (4 items; i.e., ‘I have control over how I do my work’); (3) reward—the degree to which reinforcement is consistent with expectations (4 items; i.e., ‘I receive recognition from others for my work’); (4) community—social interaction that occurs within the workplace (5 items; i.e., ‘People trust one another to fulfill their roles’); (5) fairness—the degree to which choices and resource designation are seen as reasonable and respected by others within the workplace (6 items; i.e., ‘Resources are allocated fairly here’); and (6) values—the initial drawing of individuals to a particular job through the standards and inspirations (4 items; i.e., ‘My values and organisation’s values are alike’). Responses to each statement are indicated using a 6-point Likert scale (0 = strongly disagree to 5 = strongly agree), where a total for each dimension is calculated. Previous studies have reported the internal consistency for all scales to be satisfactory, with Cronbach’s coefficient of ranging from α = 0.71–0.83 [37].

#### 2.2.2. Negative Emotional Symptoms

The Depression, Anxiety and Stress Scale (DASS-21) [38] was used to measure levels of three related emotional states (i.e., depression, anxiety, stress). The DASS-21 consists of 21 questions, with scores for each of the three emotional states derived from seven questions rated on a four-point Likert scale (0 = ‘Did not apply to me at all’ to 3 = ‘Applied to me very much or most of the time’). The depression subscale includes an assessment hopelessness, self-depreciation and a lack of interest. The anxiety subscale includes an assessment of autonomic arousal, situational anxiety, and subjective experience of anxious affect. The stress subscale includes an assessment difficulty relaxing, nervous arousal, and being easily upset [38]. As per scoring protocols, scores on each of the subscales were also doubled. A negative emotional symptoms score is computed by totaling scores for all 21 items and doubling this value. Scores above 21 for depression, 15 for anxiety and 26 for stress and above 60 for total negative emotional symptoms, are considered severe [38]. Internal consistency is high for the DASS-21, with Cronbach’s coefficient of α = 0.91, 0.80, and 0.84 for depression, anxiety and stress respectively [39].

#### 2.2.3. Psychological Wellbeing

The Warwick-Edinburgh Mental Wellbeing Scale (WEMWBS) [40] was used to measure PBW. The WEMWBS consists of 14 positively worded statements such as ‘I’ve been feeling optimistic about the future’ and ‘I’ve been dealing with problems well’, that ask officials to reflect on their everyday experiences within the last two weeks. Responses are indicated on a 6-point Likert scale (0 = ‘Never’ to 5 = ‘Very often’), with better PWB indicated by a higher score. Internal consistency is high for the WEMWBS, with a Cronbach’s coefficient of α = 0.89 [40].

### 2.3. Procedure

Participants completed an anonymous online survey using the REDCap software (Vanderbilt University, Nashville, TN, USA) [41]. Participants provided consent by clicking on the ‘I agree’ statement after reading a Plain Language Statement. The completion time of the survey was approximately 25 min duration, with surveys completed during July–September 2019. Institutional ethical approval was obtained (HEAG-H 75_2019).

### 2.4. Data Treatment and Analysis

Categorical variables were recoded as follows. Age was recoded into three categories (years; 18–30, 31–50, >50); highest level of education into two categories (high school, trade/certificate/diploma/undergraduate degree/postgraduate degree); marital status into two categories (married/committed relationship, single/divorced/separated/widowed); officiating experience into three categories (years; 0–5, 6–9, ≥10); number of sports officiated into three categories (1, 2, 3 or more); and highest level of officiating into two categories (local (community), state/national/international). Descriptive statistics (proportions) were used to summarise demographic and officiating variables. Scale/subscale scores were computed for the DASS-21, AWS and WEMWBS according to published protocols and internal consistency assessed using Cronbach’s alpha. Sample means were computed for each scale/subscale and reported against published norms. Differences in negative emotional symptoms (DASS-21 composite score) and PWB (WEMWBS) for key demographic and officiating subgroups were assessed using separate *t*-test and ANOVA analyses and magnitude of effects determined using Cohen’s d and eta-squared statistics. Associations between the two workplace measures (AWS workload and control subscales) and negative emotional symptoms and PWB were assessed using Pearson’s correlation statistic. Variables identified as significant in the univariate analyses were included in separate multivariable regression analyses predicting negative emotional symptoms and PWB. A-priori sample size estimation indicated that with α = 0.05, β = 0.20, a total sample of *n* = 200 would be required for the specified analysis. All analyses were performed using Stata Statistical Software: Release 16SE and significance of effects determined as *p* < 0.05.

## 3. Results

The final sample consisted of complete data for 317 officials (response rate 9.1%) involved in sports from different performance levels. The majority of respondents were male, over 30 years of age, were in a committed relationship, with a post-high school level of education and 10 or more years’ experience as an official. Primarily, participants focused on one sport, with rugby union, field hockey, and cricket the most popular, and officiated at local/community level with both youth and adult age groups. There was a relatively even split of paid versus volunteer officials, with the majority paid on a casual basis (see Table 1).

Internal consistency values for the AWS, DASS-21, WEMWBS scales/subscales were all acceptable [42]. Relative to normative data, officials had higher levels of workload manageability and workplace control [26] but higher levels of depression, anxiety, stress and negative emotional symptoms in relation to the general Australian population [43]; while PWB was similar to the Australian population [44] (see Table 2).

Mean negative emotional symptoms and PWB scores for key demographic and officiating subgroups are presented in Table 3. Officials who were younger (18–30 years), single/divorced/separated/widowed, less educated, and having less officiating experience had higher negative emotional symptom scores than their older, married/committed, higher educated and more experienced counterparts. There was a trend for gender (females having higher symptoms) but this effect just failed to achieve significance. For PWB, officials who were male, older (>50 years), married/committed, and higher educated had higher levels than their female, younger, single/divorced/separated/widowed and lower educated counterparts. There was a trend for officiating experience (higher levels of experience having higher levels of PWB) but this effect just failed to achieve significance. The magnitude of the effects for both measures were in the small to moderate range (see Table 3). The AWS workload and control measures were negatively correlated with negative emotional symptoms (workload: *r* = −0.33, *p* < 0.001; control: *r* = −0.37, *p* < 0.001) and positively correlated with PWB (workload: *r* = 0.29, *p* < 0.001; control: *r* = 0.48, *p* < 0.001).

Results of the separate regression analyses are presented in Table 4. Being older (>50 years) (~−7 units), having greater officiating experience (6–9 years, ≥10 years) (~−7 and −4 units respectively) and greater workload manageability (~−8 units) and workload control (~−7 units) were associated with fewer negative emotional symptoms while being single/divorced/separated/widowed (~+7) and having a high school education (~+5 units) were associated with more negative emotional symptoms. For PWB, being single/divorced/separated/widowed (~−5 units) or having a high school education (~−2 units) were associated with lower levels, while greater workload manageability (~+3 units) and workload control (~+5 units) were associated with higher levels of PWB.

## 4. Discussion

The aim of this study was to examine MH and PWB in Australian sports officials, and the factors associated with these states. Overall, officials reported high levels of negative emotional symptoms, but similar levels of PWB in comparison to the general population. Other findings indicated that officials who were younger, not in a relationship, and had less experience had higher levels of negative emotional symptoms, and those who were male, in a committed relationship, and had a higher level of education had higher levels of PWB. The ability to manage officiating workload and having control over officiating practice were strongly associated with both negative emotional symptoms and PWB; with those less able to manage their workload and with less control over decisions made in the officiating environment reporting higher levels of negative emotional symptoms and lower levels of PWB.

Negative emotional symptoms, as measured by the DASS-21, are higher than the general Australian population [43], and allowing for the low response rate and potential self-selection bias, the ‘true’ level may be even higher. A potential explanation for the observed level is the constant scrutiny from athletes, coaches and spectators while officiating, and a lack of support and education as how to best manage this. Sports officials are expected to be perfect at all times, in particular with decision making, and this increased pressure can be a further reason for the higher negative emotional symptom scores. European professional football referees have reported similar levels of negative emotional symptoms [30]. PWB scores, as measured by the WEMWBS, were similar to the general population [44] and Australian sport coaches [36], indicating that although negative emotional symptoms may be high, these officials have levels of PWB similar to the population average. Bos et al. [13] suggested that personal resources, particularly strong social support, may buffer against these high negative emotional symptoms. An explanation for the combined high negative emotional symptoms score and the average PWB score can be based on the general consensus that MH and PWB are independent constructs [2], supporting the application of the two continua model of mental illness and health [12], and therefore highlighting the need to measure both MH and PWB to fully understand the impact on sports officials. Gorczynski and Webb [3] noted that with little known regarding MH of sports officials, it is not possible to identify how negative emotional symptoms actually affect the individual. Officials also reported a higher ability to manage workload and higher levels of workplace control than general population norms [26]. Both of these factors are known to increase job satisfaction, which can mediate the effects of higher negative emotional symptoms and improve PWB. Similar findings have been made in academic populations [45] and corporate settings [46]. Further, potentially these officials are proactive in coping with negative emotion symptoms early, concentrating on diet and exercise to enhance officiating performance, or talking to a positive support network of family, friends, colleagues or professionally trained consultants. While many organisations have developed strategies to recruit and retain officials, further investment into campaigns promoting respect and tolerance of sport officials may also be required.

Supporting Hypothesis 1, the findings of the current study highlight the association between age and MH and PWB. Older officials had lower negative emotional symptoms. Potentially they do not experience the same levels of abuse that younger officials do, or they have become more experienced at managing their environment. Alternatively, negative emotional symptoms decrease with age and these officials are better at self-regulating [47]. Similar findings have been identified in sports coaches [36]. One suggestion is that older officials are more intrinsically motivated than younger officials, which could moderate the stressors associated with the role [33]. However, there are other factors such as the adoption of more effective coping skills or increased social support available for older officials. Mental toughness is known to increase with age and experience [14], with mental toughness negatively correlated with depression, anxiety and stress [48]. Slack et al. [49] identified mentally tough sports officials utilise more adaptive coping skills, by drawing on past experience, which strengthens the possibility that this construct may, in part, explain the relationships between age and experience, and better MH and PWB. Lin et al. [50] have identified similar findings in a range of education and workplace settings.

Officials who were married or in a committed relationship had significantly better MH and PWB, supporting Hypothesis 2, potentially as a result of the social support provided by a significant other helping to reduce negative emotional symptoms. The availability of someone to regularly debrief in a supportive manner could alleviate the demands of officiating. Organisations supporting officials may benefit from providing support networks for all officials as a means to manage the stressors of officiating and promote increases in PWB. Seeking social support from those close is a frequently used coping strategy of sports officials [31]. Family have been acknowledged as a strong motivational factor for participation as a sports official [51] and as such encouraging officials to build their social relationships around officiating practice may promote better PWB. Similar findings have been made in the organisational psychology literature [52]. Officials in the current study who completed higher levels of education had significantly better MH and PWB. For officials a higher education level may afford them greater levels of self-awareness and self-regulation increasing their ability to cope with the known stressors of officiating.

In general, the officials reported a good ability to manage their workload and not allow personal or career aspects to influence their officiating practice. Officials reporting a higher ability to balance workload commitments scored significantly lower in negative emotional symptoms and higher in PWB, supporting Hypothesis 3. Having higher levels of PWB may allow officials to make better decisions and perform at their peak, and as such both the individual official themselves and their supporting association are encouraged to identify strategies to maintain an officiating-life balance. Auger et al. [53] and Voight [31] noted the prime causes of stress for officials were managing conflicts between officiating, family and employment. An inability to do this has the potential to negatively impact officials’ PWB. Most officials participate not as a primary occupation or as a means of generating meaningful income, but rather as a form of enjoyment [51]. This demonstrates a willingness to commit free time to the activity, but officials can be further supported in this. Cuskelly and Hoye [1] suggested flexible scheduling may be essential in assisting them to manage their own MH and PWB. This approach has been successful in the healthcare professions [54] and general organisational psychology literature [46]. Practically this may be difficult due to limited numbers of registered officials but may be an important component of managing negative emotional symptoms. The addition of suitable rewards may also be of benefit for officials and act as a moderator against negative emotional symptoms.

Officials reported generally high levels of control over the decisions needed to be made in the workplace. Supporting Hypothesis 4, higher control is associated with better MH and better PWB. Officials who indicated greater control are more likely to understand the expectations of their role and have full knowledge of their duties. Having role clarity will assist officials in developing greater control and therefore assist in enhancing MH and PWB. A lack of sufficient training in managing known stressors, such as verbal abuse, could also impact MH and PWB of these officials [55]. While not directly measuring workplace support in the present study, the relationship between control and MH and PWB suggests the need for organisational support for officials. The healthcare industry recommends regular discussion by management and employees on this [52]. Cuskelly and Hoye [1] advocated for greater organisational support to manage officiating stressors. While Livingston and Forbes [56] called for officiating organisations to provide opportunities for advancement, they also noted a lack of transparency in the officiating progression process can initiate negative emotional states.

To our knowledge this is the first study examining MH and PWB among a large sample of Australian sports officials. The application of validated work engagement, MH and PWB measures allows for comparison with general population levels and other similar groups. At the same time, several limitations are noted. The cross-sectional study design enables reporting on associations, not causation, nor does it permit analysis of, or controlling for, change in mental health and wellbeing, as well as time-varying covariate. The study response rate was low and limits scope for strong, generalisable, conclusions. Generalisability of the findings is further limited to one country at one specific time point (potentially as sport is seasonal some officials may not have officiated recently). The recruitment of current, practicing officials meant there was a survival bias and that it was not possible to report on officials who have withdrawn from officiating. Other factors important for MH and PWB, such as social support and personality, were not measured and not available for the present analysis. Replication of the present study is recommended. Such studies should employ more rigorous recruitment strategies to increase response rates and maximise sample size as well as draw from a wider demographic base (i.e., officials <18 years, officials who have recently withdrawn) to also strengthen generalizability. These studies should also incorporate measures for other factors (e.g., social support, personality) important for MH and PWB. Complimentary studies that utilise a mixed method approach will enable a more nuanced understanding of sports officials’ MH and PWB.

## 5. Conclusions

Generally, officials in this study showed higher negative emotional symptoms in comparison to the general population but similar PWB. A number of factors were identified to be associated with lower/higher levels MH and PWB. The ability to manage officiating workloads and to demonstrate professional autonomy significantly influence the MH and PWB of officials. Sporting organisations, including bodies that represent the interests of officials, should consider ways to enable and value perspectives of officials in the development of fixturing and rostering and also payment (where possible to defray the need to balance up work and other personal roles and responsibilities) as well as broader sport-related issues such as strategies to promote respect and tolerance for the important role played by officials.

## Figures and Tables

**Table 1:** Demographic characteristics of sample.

Variable	*n*	%
*Gender*		
Male	244	77.0
Female	73	23.0
*Age group (years)*		
18–30	64	20.2
31–50	114	36.0
>50	139	43.8
*Relationship status*		
Married/committed relationship	229	72.2
Single/divorced/separated/widowed	88	27.8
*Highest level of education*		
High school	96	30.3
Trade/certificate/diploma/undergraduate/postgraduate	221	69.7
*Number of sports officiated*		
One	268	84.5
Two	37	11.7
Three or more	12	3.8
*Sport(s) officiated #*		
Rugby union	94	-
Field hockey	57	-
Cricket	56	-
Netball	35	-
Rugby league	27	-
Australian Rules football	23	-
Soccer	21	-
All other sports (e.g., basketball, boxing, athletics, lacrosse)	77	-
*Highest level of competition officiated*		
Local/community	217	68.5
State	61	19.2
National	25	7.9
International	14	4.4
*Engagement*		
Volunteer	172	54.3
Paid	145	45.7
*Nature of paid engagement*		
Full time	50	34.5
Part time	23	15.9
Casual	72	49.7
*Age groups usually officiated*		
Children/youth (up to 16 years)	21	6.6
Adults/senior (over 16 years)	105	33.1
Both	191	60.3
*Gender usually officiated*		
Males	113	35.6
Females	26	8.2
Both	178	56.2
*Officiating experience (years)*		
0–5	73	23.0
6–9	61	19.2
≥10	183	57.7

# multiple responses across sports.

**Table 2:** Scale reliability (Cronbach’s alpha), descriptive statistics and comparative normative data.

Variable	*α*	*M* (*SD*)	*95% CI*	*Norms* (*M*)
*AWS*				
Workload	0.73	3.2 (0.8)	3.1, 3.3	2.83 [26]
Control	0.82	3.8 (0.8)	3.7, 3.8	3.35 [26]
*DASS-21*				
Depression	0.92	7.3 (8.6)	6.4, 8.3	5.14 [43] *
Anxiety	0.82	4.9 (6.1)	4.2, 5.6	3.48 [43] *
Stress	0.88	10.4 (8.2)	9.4, 11.3	7.98 [43] *
Negative emotional symptoms	0.94	22.6 (20.8)	20.3, 24.8	16.60 [43] *
*WEMWBS*				
Total psychological wellbeing score	0.95	53.3 (9.7)	52.2, 54.3	52.3 [44]

* Reported normative scores have been double in accordance to scoring protocol. *α* is the Cronbach’s alpha scores.

**Table 3:** Negative emotional symptoms (DASS-21 composite) and psychological wellbeing (WEMWBS total score) for officials’ subgroups.

Variable	*Negative Emotional Symptoms*				*Psychological Wellbeing*			
	*M* (*SD*)	*t*/*F*	*p*	*d*/*η*^2^	*M* (*SD*)	*t*/*F*	*p*	*d*/*η*^2^
Demographic								
*Gender*								
Male	21.3 (21.0)	−1.91	0.06	0.26	**54.2 (9.6)**	**3.05**	**0.003**	**0.41**
Female	26.6 (19.5)				**50.3 (9.3)**			
*Age group (years)*								
18–30	**31.8 (24.7)**	**9.3**	**<0.001**	**0.06**	**49.3 (10.3)**	**9.3**	**<0.001**	**0.06**
31–50	**22.2 (19.7)**				**52.9 (9.3)**			
>50	**18.6 (18.3)**				**55.4 (9.0)**			
*Relationship status*								
Married/committed relationship	**20.3 (19.2)**	**−** **2.9**	**0.005**	**0.38**	**54.9 (9.3)**	**5.1**	**<0.001**	**0.64**
Single/divorced/separated/widowed	**28.4 (23.5)**				**48.9 (9.4)**			
*Highest level of education*								
High school	**26.2 (22.0)**	**2.1**	**0.04**	**0.25**	**51.5 (9.7)**	**−2.2**	**0.03**	**0.26**
Trade/certificate/diploma/undergraduate/postgraduate	**21.0 (20.0)**				**54.0 (9.6)**			
Officiating								
*Highest competition level*								
Community	23.2 (21.7)	0.90	0.37	0.03	53.5 (10.0)	0.48	0.63	0.06
State/national/international	21.1 (18.5)				52.9 (8.9)			
*Engagement*								
Volunteer	22.9 (21.8)	00.30	0.76	0.03	53.1 (9.8)	−0.45	0.65	0.05
Paid	22.2 (19.6)				53.5 (9.6)			
*Officiating experience (years)*								
0–5	**29.6 (25.5)**	**5.6**	**0.004**	**0.03**	50.9 (10.2)	2.8	0.06	0.02
6–9	**20.1 (17.2)**				53.9 (9.2)			
≥10	**20.6 (19.2)**				54.0 (9.5)			

*t/F* denotes the *t* value/*F*; *d*/*η*^2^ denotes Cohen’s d/Eta squared effect size statistics; The bold text helps identify the significant results.

**Table 4:** Multivariate models predicting negative emotional symptoms (DASS composite score) and psychological wellbeing (WEMWBS total score).

Variable	*Negative Emotional Symptoms*			*Psychological Wellbeing*		
	*b*	*se*	*p*	*b*	*se*	*p*
Gender, female	−2.19	2.56	0.39	−0.16	1.12	0.89
Age (ref: 18–30 years)						
31–50 years	−5.00	3.61	0.17	0.22	1.38	0.87
>50 years	**−7.22**	**3.68**	**0.05**	2.23	1.46	0.12
Relationship status; single/divorced/separated/widowed	**7.00**	**2.76**	**0.01**	**−5.48**	**1.09**	**<0.001**
Highest education; high school	**5.48**	**2.22**	**0.01**	**−2.36**	**0.95**	**0.01**
Officiating experience (ref: 0–5 years)						
6–9 years	**−7.15**	**3.34**	**0.03**	1.70	1.41	0.23
≥10 years	**−4.43**	**2.94**	**0.13**	0.65	1.15	0.57
AWS workload	**−7.60**	**1.41**	**<0.001**	**2.78**	**0.57**	**<0.001**
AWS control	**−6.70**	**1.38**	**<0.001**	**4.67**	**0.61**	**<0.001**

‘*b*’ refers to the unstandardised regression coefficient; ‘*se*’ refers to the standard error of ‘*b*’; The bold text helps identify the significant results.

## Supplementary Materials

The following are available online at https://www.mdpi.com/1660-4601/17/21/8265/s1, Table S1: Summary of study variables, measures and example survey questions.
