# Supplementary material for: Examining Negative Emotional Symptoms and Psychological Wellbeing of Australian Sport Officials
Source: Int J Environ Res Public Health. 2020 Nov 9;17(21):8265. doi: 10.3390/ijerph17218265 (PMC7664859; doi:10.3390/ijerph17218265)
Supplement: Supplementary file 1 [file ijerph-17-08265-s001.pdf]

Supplementary Table 1. Summary of study variables, measures and example survey questions

Variables	*Measure*	*Example questions*
Demographics	Gender	What is your gender?
	Age group (years)	How old are you?
	Relationship status	What is your marital status?
	Highest level of education	What is your highest level of education?
Officiating	Sport(s) officiated	What sport/s do you officiate in?
	Current officiating role	What is your current role in [sport]?
	Level of competition officiated (for each sport)	What level of competition do you usually officiate [sport] in?
	Age groups usually officiated	What age group/s do you usually officiate?
	Gender usually officiated	What group/s do you usually officiate?
	Nature of paid engagement	What is the nature of your officiating?
	Officiating experience (years)	How many years have you been an official for?
Workload	Areas of Worklife Scale (AWS) – Workload Scale	I work intensely for prolonged periods of time.<br>I have enough time to do what is important.
Control	Areas of Worklife Scale (AWS) – Control Scale	I can influence management to obtain equipment and space I need.<br>I have professional autonomy / independence
Negative Emotional Symptoms	Derived from Depression, Anxiety and Stress Scale (DASS-21)	
	- Depression scale	I couldn't seem to experience any positive feelings at all.<br>I felt like I had nothing to look forward to.
	- Anxiety scale	I was aware of dryness in my mouth.<br>I experienced breathing difficulty.
	- Stress scale	I found it hard to wind down.<br>I tended to over-react to situations.
Psychological Wellbeing	Warwick-Edinburgh Mental Wellbeing Scale (WEMWBS) – total score items	I've been feeling relaxed.<br>I've been feeling good about myself.
